# Increased Duration of Heating Boosts Local Drug Deposition during Radiofrequency Ablation in Combination with Thermally Sensitive Liposomes (ThermoDox) in a Porcine Model

**DOI:** 10.1371/journal.pone.0139752

**Published:** 2015-10-02

**Authors:** Christine E. Swenson, Dieter Haemmerich, Donald H. Maul, Bridget Knox, Nicole Ehrhart, Robert A. Reed

**Affiliations:** 1 Celsion Corporation, Lawrenceville, NJ, United States of America; 2 Medical University of South Carolina, Charleston, SC, United States of America; 3 PreClinical Research Services, Inc., Fort Collins, CO, United States of America; 4 Colorado State University Animal Cancer Center, Fort Collins, CO, United States of America; Academia Sinica, TAIWAN

## Abstract

**Introduction:**

Radiofrequency ablation (RFA) is used for the local treatment of liver cancer. RFA is effective for small (<3cm) tumors, but for tumors > 3 cm, there is a tendency to leave viable tumor cells in the margins or clefts of overlapping ablation zones. This increases the possibility of incomplete ablation or local recurrence. Lyso-Thermosensitive Liposomal Doxorubicin (LTLD), is a thermally sensitive liposomal doxorubicin formulation for intravenous administration, that rapidly releases its drug content when exposed to temperatures >40°C. When used with RFA, LTLD releases its doxorubicin in the vasculature around the zone of ablation-induced tumor cell necrosis, killing micrometastases in the ablation margin. This may reduce recurrence and be more effective than thermal ablation alone.

**Purpose:**

The purpose of this study was to optimize the RFA procedure used in combination with LTLD to maximize the local deposition of doxorubicin in a swine liver model. Pigs were anaesthetized and the liver was surgically exposed. Each pig received a single, 50 mg/m^2^ dose of the clinical LTLD formulation (ThermoDox®). Subsequently, ablations were performed with either 1, 3 or 6 sequential, overlapping needle insertions in the left medial lobe with total ablation time of 15, 45 or 90 minutes respectively. Two different RFA generators and probes were evaluated. After the final ablation, the ablation zone (plus 3 cm margin) was dissected out and examined for doxorubicin concentration by LC/MS and fluorescence.

**Conclusion:**

The mean Cmax of plasma total doxorubicin was 26.5 μg/ml at the end of the infusion. Overall, increased heat time from 15 to 45 to 90 minutes shows an increase in both the amount of doxorubicin deposited (up to ~100 μg/g) and the width of the ablation target margin to which doxorubicin is delivered as determined by tissue homogenization and LC/MS detection of doxorubicin and by fluorescent imaging of tissues.

## Introduction

### Lyso-thermosensitive liposomal doxorubicin (LTLD)

The concept of enhanced local release of drug by hyperthermia using thermally sensitive liposomal carriers was introduced nearly 4 decades ago [1]. In that publication, five mechanisms for enhancing drug delivery were considered; a) promotion of thermal drug release at temperatures near the lipid bilayer phase transition; b) increased perfusion of the target area; c) increased capillary uptake of particles (enhanced permeability and retention, EPR) effect; d) increased susceptibility of the cell to the drug mechanism of action; and e) increased endocytosis. Exploitation of the first mechanism was realized with a DPPC (1,2-Dipalmitoyl-sn-glycero-3-phosphatidylcholine)/DSPC (distearoyl phosphatidylcholine) (3:1, mol:mol) liposome which demonstrated a maximum rate of release at ~ 45°C. More recently, a new lyso-thermosensitive liposome platform was introduced [2] which incorporated a lyso-lipid into a DPPC liposome membrane to effectively shift the maximum release rate down into the 40–43°C, window, and thus allowing promotion of thermal drug release at temperatures as low as 40°C.

Importantly, this type of formulation facilitates rapid release within a few seconds [3]. Several recent studies suggest triggered intravascular release (TIR) to be the dominating delivery mechanism with this type of rapid-release formulation, and the reason for the high efficacy of this delivery mechanism [4–7].

Lyso-thermosensitive liposomal doxorubicin (ThermoDox^®^), as used in the current study, is a heat-activated liposomal formulation of doxorubicin that facilitates targeted delivery to tumor/tissue volumes at temperatures exceeding 40°C. The active ingredient of LTLD (doxorubicin hydrochloride) is a cytotoxic anthracycline that is a well-established chemotherapeutic that continues to be the standard of care for the treatment of hematological malignancies, many types of carcinomas, soft tissue sarcomas, and is often used in combination regimens. The drug product, LTLD, envelopes doxorubicin in liposomes that are made from three synthetic phospholipids: DPPC, MSPC (1-Stearoyl-2-hydroxy-sn-glycero-3-phosphatidylcholine) and DSPE-MPEG2000 (1,2-Distearoyl-sn-glycero-3-phosphoethanolamine-N-methoxypoly-ethyleneglycol 2000), a membrane composition that provides the unique thermal and rapid triggered release of doxorubicin. LTLD is designed to provide: 1) near complete encapsulation of doxorubicin HCl, 2) rapid release of the encapsulated doxorubicin with mild thermal warming (>40°C), and 3) the ability to provide adequate systemic circulation to allow the use of heat inducing medical devices to warm the target tumor, initiating a rapid drug release in the targeted tumor vasculature. It is believed that if high concentrations of doxorubicin can be delivered locally, greater amounts of drug will be available to enhance tumoricidal activity without increasing systemic toxicity.

### Radiofrequency Ablation (RFA)

Radiofrequency ablation (RFA) is a locoregional thermal therapy, and is clinically used to treat unresectable tumors of the liver, as well as in other organs such as the kidney, lung and bone [8]. A needle like-electrode is placed percutaneously, laparoscopically or during open surgery in the tumor with image guidance. Radiofrequency electric current is applied to the tissue adjacent to the electrode and causes localized tissue heating and results in coagulation necrosis at temperatures above ~50°C [9]. Generally, the ablation target includes a 1 cm margin around the tumor to ensure complete thermal destruction of the tumor [10]. However, this cannot always be accomplished due to the location of the tumor or the heat-sink effect of the extensive vascularity of the liver, the limitations of existing probe designs and intra-operative imaging and because often tumor microsatellites exist distant from the macroscopic tumor [11]. For larger liver tumors (>3 cm diameter), the local failure rate for RFA can be greater than 40%, presumably due to untreated cancer cells found in the margin around the ablation zone [9, 12–14]. LTLD used in combination with ablative procedures takes advantage of a tissue volume that surrounds the zone of ablation where the tissue is still well perfused and viable, and the tissue temperature exceeds 40°C, creating the required combination to allow LTLD to release its therapeutic payload. The LTLD treatment approach is designed to target the delivery of high concentrations of doxorubicin to these tumor margins of ablative treatments utilizing a single treatment given concurrently with a thermal ablative procedure. Therefore, the overall hypothesis is that LTLD used in combination with ablative treatments will lead to an improved therapy for the local treatment of primary liver cancer [10].

The pharmacokinetics and metabolism of conventional doxorubicin HCl have been well characterized. An intravenous bolus dose of doxorubicin in animals and humans produces initial high plasma concentrations that decrease rapidly due to extensive distribution into systemic tissues with an initial plasma half-life of 4.8 min (T ½α) to 2.6 hr (T½ β). The volume of distribution in humans ranges from 700–1100 L/m^2^, plasma clearance values range from 24 to 35 L/h/m2. Renal clearance is low (~12%) and biliary excretion is high (>50%) [15]. In contrast, the clearance of total (i.e., encapsulated and unencapsulated) doxorubicin after a 30 minute iv infusion of LTLD was 1.1 L/h/m^2^ and the volume of distribution was 5.1 L/m^2^ [16] indicating that plasma total doxorubicin levels after LTLD are higher and more prolonged than after conventional drug. This allows a 3–4 hour window during which thermal energy can be applied to the target tissue to trigger the intravascular release of doxorubicin from the liposome. Although the plasma total doxorubicin levels after LTLD are higher and more prolonged than after doxorubicin, the levels are not as high and prolonged as another commercial liposomal formulation of doxorubicin, Doxil®, and palmar-plantar erythrodysesthesia (‘hand-foot syndrome”) has not been reported with LTLD.

### In vivo swine study

The purpose of this study was to visualize and optimize the delivery of the anticancer drug doxorubicin when administered in thermally sensitive liposomes in combination with radiofrequency ablation (RFA) for the treatment of liver tumors. Based on a post-hoc analysis of a sub-group of patients in a phase III study of LTLD plus RFA in hepatocellular carcinoma, it was found that longer duration heating improves outcomes [17]. Therefore, in this study, parameters such as ablation duration (as determined by the number of sequential, overlapping ablations) and timing of the ablation in relation to the infusion of the drug were evaluated. In vivo studies of normal liver of pigs were performed because of a lack of large animal liver tumor model and because these livers are of sufficient size to allow for use of clinically relevant radiofrequency generators and ablation probes. The in vivo normal pig liver is an accepted model for the study of clinical RFA devices and methods [18, 19].

## Material and Methods

### Materials

The Lyso-Thermosensitive Liposomal Doxorubicin (ThermoDox) was provided by Celsion Corporation as a frozen solid. After thawing, each vial contained 15 ml of liposomal dispersion with 2 mg/ml doxorubicin HCl.

### Study Design

The study design and animal usage were reviewed and approved by the PreClinical Research Services, Inc. (Fort Collins, CO) Institutional Animal Care and Use Committee (IACUC) for compliance with regulations prior to study initiation (IACUC 1326). Animal welfare, housing and research procedures for this study were conducted in compliance with all federal animal welfare laws, policies, and regulations, including the U.S. Department of Agriculture’s (USDA) Animal Welfare Act, the Guide for the Care and Use of Laboratory Animals, the Guide for the Care and Use of Agricultural Animals in Research and Teaching, Public Health Service National Institutes of Health (PHS-NIH) Office of Laboratory Animal Welfare (OLAW), and the American Veterinary Medical Association (AVMA) Guidelines for Euthanasia.

Twenty two (22) female domestic pigs, 3–6 months old, mean weight 54.9 kg, from Midwest Farms, Burlington, CO were used for this study. Animals were housed in socially compatible groups with ≥10 sq. ft. per animal.

Each animal was fasted at least 12 hours prior to surgery with free access to water. Pigs are inherently sensitive to liposome-induced hypersensitivity reactions [20], so they were premedicated with steroids and antihistamines prior to anesthesia (described in S1 Table).

After anesthesia, ground pads were placed appropriately on the animal’s thighs. The carotid artery was cannulated for pressure measurement. A jugular venous line was placed for venous blood sampling. Auricular veins were catheterized with one being used for administration of intravenous fluids with a second venous line placed for drug administration. The liver was surgically exposed. Intraoperative ultrasonic examination of the liver was performed to locate appropriate sites with relatively few blood vessels for ablation. The ablation devices used were a Covidien Valley Lab (“cool tip”, model #CTRF117) generator with a single needle electrode (ACT1510; exposure 1.0 cm, length 15 cm) or an Angiodynamics (RITA, Model # 1500x) generator with Starburst XL (cat # 700–101930; 10 cm length) multi-tine electrode with tines extended to 2 cm. At time 0, the LTLD infusion (1.43 mg/kg diluted in 250 ml D5W [equivalent to 50 mg/m^2^]) began, and was infused over 30 min. The mean dose administered was 95.4% of the intended dose.

Study A involved 3 pigs. Each pig received a single dose of LTLD and had 4 carefully timed ~ 1 cm non-overlapping ablations (each in a different lobe starting at 15 min [right lateral], 45 min [right medial], 75 min [left medial], and 105 min [left lateral] after the start of the LTLD infusion). Each ablation (with the Covidien device) was targeted to last 12 minutes with 3 minutes cool down following each ablation. Blood samples (approximately 4.0 ml with K2-EDTA as anticoagulant) were collected from all animals at predose, 0.25, 0.5, 0.75, 1.0, 1.5, 2.0 and 2.25 hours post start infusion. Plasma was separated in a refrigerated centrifuge within 30 minutes and then frozen (-70°C) until shipped to the bioanalytical lab. At 15 minutes after the end of the last ablation (~2.25 hr or 135 minutes after the start of the infusion), the animal was euthanized (pentobarbital, >88 mg/kg IV) and each ablation zone (plus a 3 cm margin outside the ablation zone) was visually identified and dissected out whole, weighed and frozen (-70°C) for determination of the total amount of doxorubicin in the ablated and margin region. A separate, non-ablated sample of liver tissue was collected, weighed and frozen for determination of the total amount of doxorubicin (“remote” sample).

Studies B and C each involved 3 groups of 3 pigs each. Each pig received a single dose of LTLD and had a single ablation in the left medial liver lobe (starting 15 minutes after the start of the LTLD infusion) using 1, 3 or 6 sequential, overlapping ablations. According to the manufacturer specifications, each ablation was targeted to last 12 minutes with the generator ON and 3 minutes for cool-down between each ablation using Covidien (study B) or Angiodynamics (study C) radiofrequency generators and probes. At 15 minutes after the final ablation, each animal was euthanized and the ablation zone including a 3 cm margin was dissected out and 2 slices (~2 cm thick each) through the center of the ablation zone and perpendicular to the electrode track(s) were obtained. The first slice was frozen and stored for later fluorescence imaging and the second slice was further dissected to obtain six 2 cm by 0.8 cm punch biopsy samples (each weighing approximately 0.5 g) radiating out from the ablation zone in two directions (A, B) or six directions (A-F). The punch biopsy closest to the ablation zone was designated location 1 and that furthest from the ablation zone was designated location 6 (S1 Fig). The biopsy samples were homogenized and analyzed for doxorubicin concentration by LC/MS.

### Bioanalytical Methods

Plasma total doxorubicin and its respective stable label internal standard ([^13^C] Doxorubicin–d3) were released from swine plasma/liver-saline homogenate by protein precipitation and the supernatant was chromatographed using reversed phase HPLC with a Gemini C6 –Phenyl analytical column with tandem mass spectrometry (MS/MS) detection. The quantitation range was 100–50,000 ng/ml (plasma) or 0.100 to 50.0 mcg/g (liver tissue). The precision and accuracy (including 2x and 5x dilutions) were within 20% of nominal concentrations of quality control samples.

### Fluorescence Imaging

Imaging studies were performed with a dedicated fluorescence imaging system (Maestro 2, Perkin-Elmer). For all imaging studies, green light at 520 nm was used for excitation, and emission spectra were measured between 560–720 nm (10 nm steps). The frozen liver slices were thawed, and the tissue side where the ablation was visible imaged at 1 s exposure time, or alternatively at 0.5 s exposure time in case of signal saturation. Spectral unmixing was performed based on reference doxorubicin and tissue-spectra acquired earlier, and doxorubicin signal intensity was determined. In addition, each sample was imaged photographically with white light illumination to depict the ablation zone. A calibration curve was created based on liver samples spiked with known, varying amounts of doxorubicin (measured using LC/MS-MS) and the fluorescence of those reference samples. There was an approximately square relationship between fluorescence and doxorubicin concentration, similar to prior published studies [21]. Image analysis was done using ImageJ version 1.49 (National Institutes of Health). A histogram of the pixel intensities in the region of interest of each image was generated and the intensity values were converted to concentrations based on the calibration curve. The total area above 10 μg/g doxorubicin (twice the concentration found in “remote” samples taken from areas of the liver not near ablation zones) was calculated and compared for the two devices and ablation times with significance (p <0.05) determined by the two sample F-test for variances (Excel).

## Results

### LTLD administration

LTLD was well tolerated in 14 pigs. It is well known that pigs often present anaphylactic reactions following administration of particulate drugs such as liposomes [20]. Although all pigs received pre-medication, seven pigs had mild to severe anaphylactic reactions shortly after the start of the LTLD infusion, which presented as hyperemia and hypertension or hypotension. Two animals responded well to treatment with epinephrine and internal cardiac massage, one animal with phenylephrine, two animals responded well to splanchnic massage alone, and two animals recovered with no treatment. One animal had a severe anaphylactic reaction during the infusion that presented with bradycardia and ventricular tachycardia, which went into coarse ventricular fibrillation. The animal was treated with epinephrine, atropine, lidocaine, external chest compressions and a precordial thump, but arrested before the end of the infusion.

### Swine plasma pharmacokinetics and effect of time of ablation in relation to the LTLD infusion

**Fig 1:**
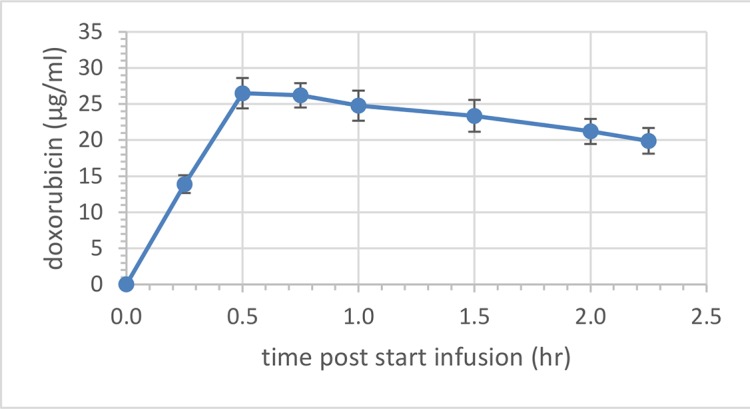
Mean (± SEM) plasma concentration of total doxorubicin in pigs in Study A. Pigs (n = 3) received a single, intravenous infusion of LTLD (1.43 mg/kg) over 30 minutes.

Total plasma doxorubicin for the 3 pigs in study A (each getting 4 sequential, non-overlapping ablations after a 1.43 mg/kg or 50 mg/m^2^ dose of LTLD) is shown in Fig 1. The mean peak plasma concentration (at the end of infusion) was 26.5 μg/ml (which is similar to the Cmax observed in humans receiving a 50 mg/m^2^ dose of LTLD [16]) and the estimated half life was 4.83 hours.

In study A, the entire ablation volumes and a 3 cm margin were homogenized and assayed for total doxorubicin concentrations. The actual measured concentrations did not show any significant differences (by single factor analysis of variance) between ablations that were initiated at 15, 45, 75 or 105 minutes after the start of the LTLD infusion and all samples were comparable to or only slightly lower (~3 μg/g) than concentrations found in the “remote” biopsies (~5 μg/g). This does not preclude the possibility that the time of the ablation in relation to the drug infusion is important to the optimal delivery of doxorubicin to the target site but suggests that the samples were too large (and included the actual ablation zone where the drug concentration is close to zero) so that any targeted area was diluted with non-targeted tissue.

### Effect of ablation duration on tissue concentrations of doxorubicin

**Fig 2:**
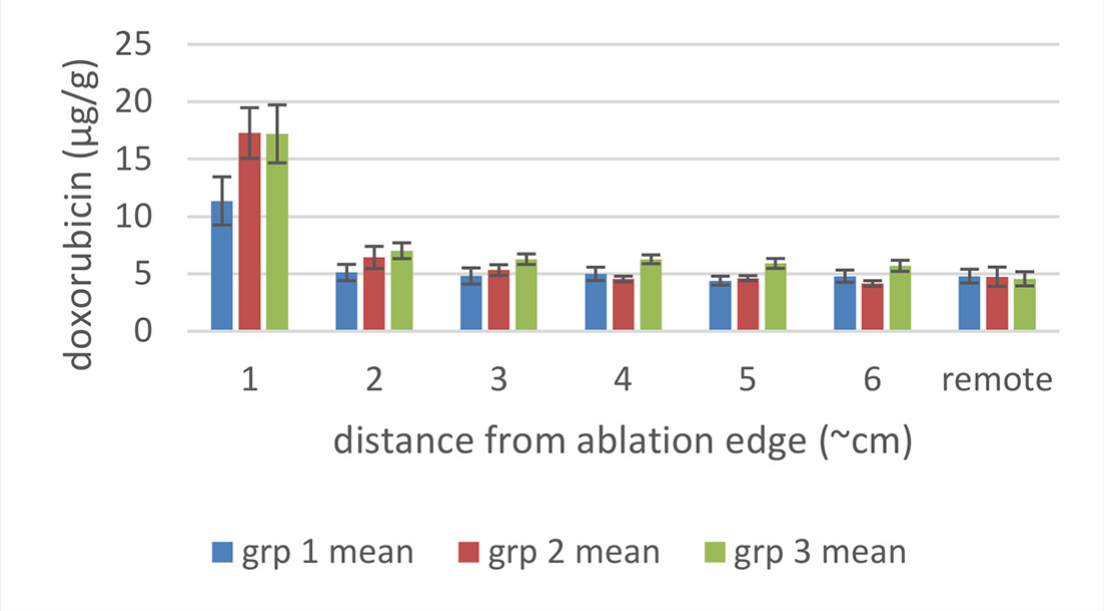
Mean (± SEM) doxorubicin tissue concentrations around the ablation zones of pigs in Study B. Punch biopsies were collected radiating out from the liver ablation zone after 1 (group 1, n = 3), 3 (group 2, n = 3) or 6 (group 3, n = 3) sequential, overlapping ablations using the Covidien device. Distance 1 is just adjacent to the ablation margin and distance 6 is the furthest away from the ablation margin.

In Study B, animals received either a single 12 minute ablation and 3 minutes cool down (group 1) three sequential, overlapping 12 minute ablations (group 2) or six sequential, overlapping 12 minute ablations (group 3) using a Covidien RF generator and single cooled needle electrodes. Biopsies from liver tissue remote from the ablation site had doxorubicin concentrations (as determined after homogenization and assay by LC-MS/MS) of about 5 μg/g. As expected, those biopsies taken closest to the edge of the ablation had the highest mean concentrations of doxorubicin in all groups (Fig 2).

For group 1 (n = 3, with an actual mean ablation time of 13 minutes) the average doxorubicin tissue concentration in the ablation zone margin was 11.0 μg/g (Fig 2, 1 cm distance), with the remaining locations (≥ 2 cm distant) showing the same doxorubicin concentration as the remote sample.

For group 2 (n = 3 with a mean actual ablation time of 39 minutes), the average doxorubicin tissue concentration in the ablation zone margin was 17.0 μg/g with slightly increased concentration also at 2 and 3 cm distance (Fig 2) with the remaining locations (> 3 cm distant) showing the same concentration as the remote sample.

For group 3 (n = 3 with a mean actual ablation time of 73 minutes) concentrations were similar to group 2.

Overall, the increase in the number of ablations and cumulative heating duration resulted in a significant increase in concentration in the ablation margin between 13 and 39 minutes, but with no significant difference between 39 and 73 minutes.

**Fig 3:**
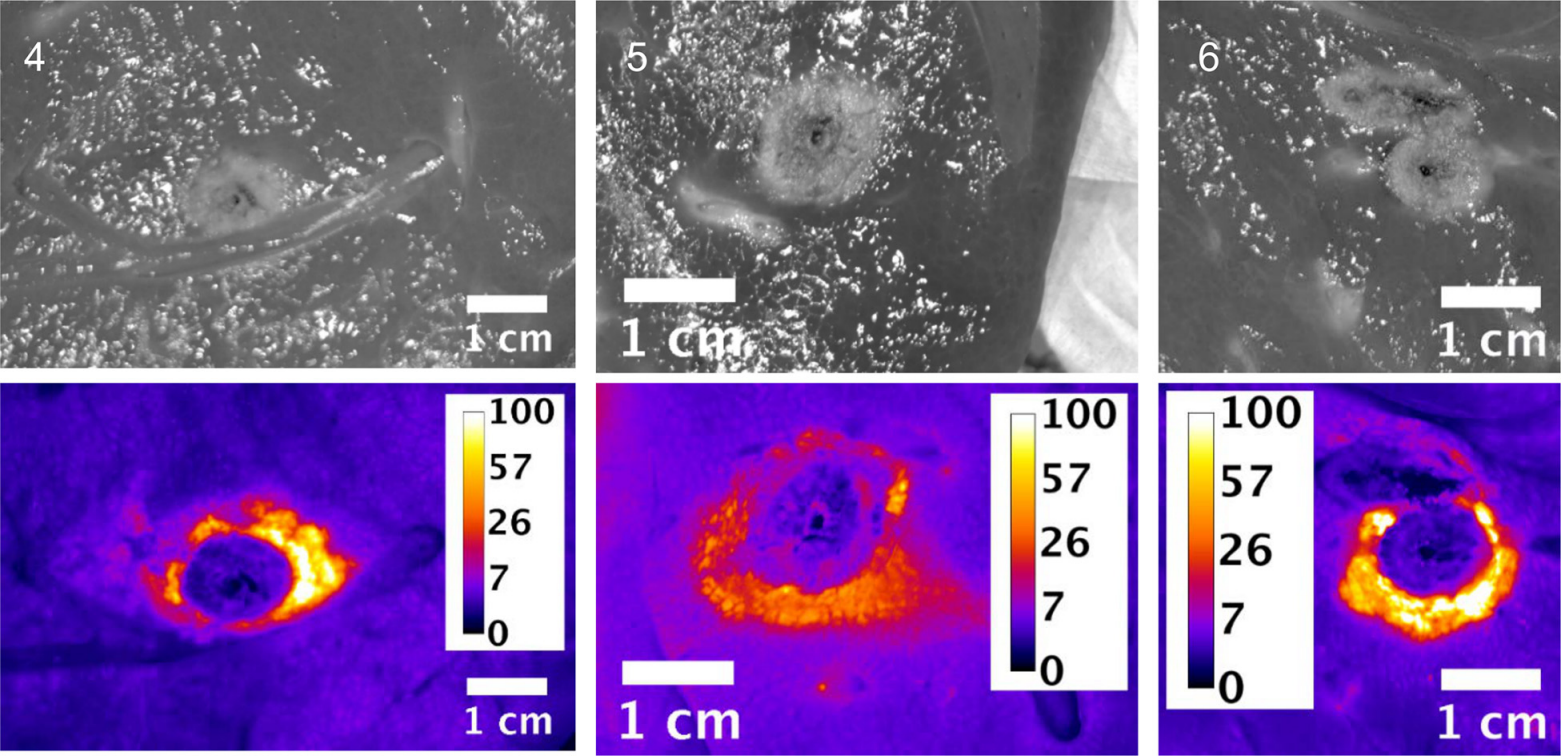
Plain and fluorescent images of ablation zone in pigs in Study B, group 1. Each pig received a single 12 minutes ablation and 3 minute cool down (total time = 15 minutes) with the Covidien device. Scale is μg/g doxorubicin.

**Fig 4:**
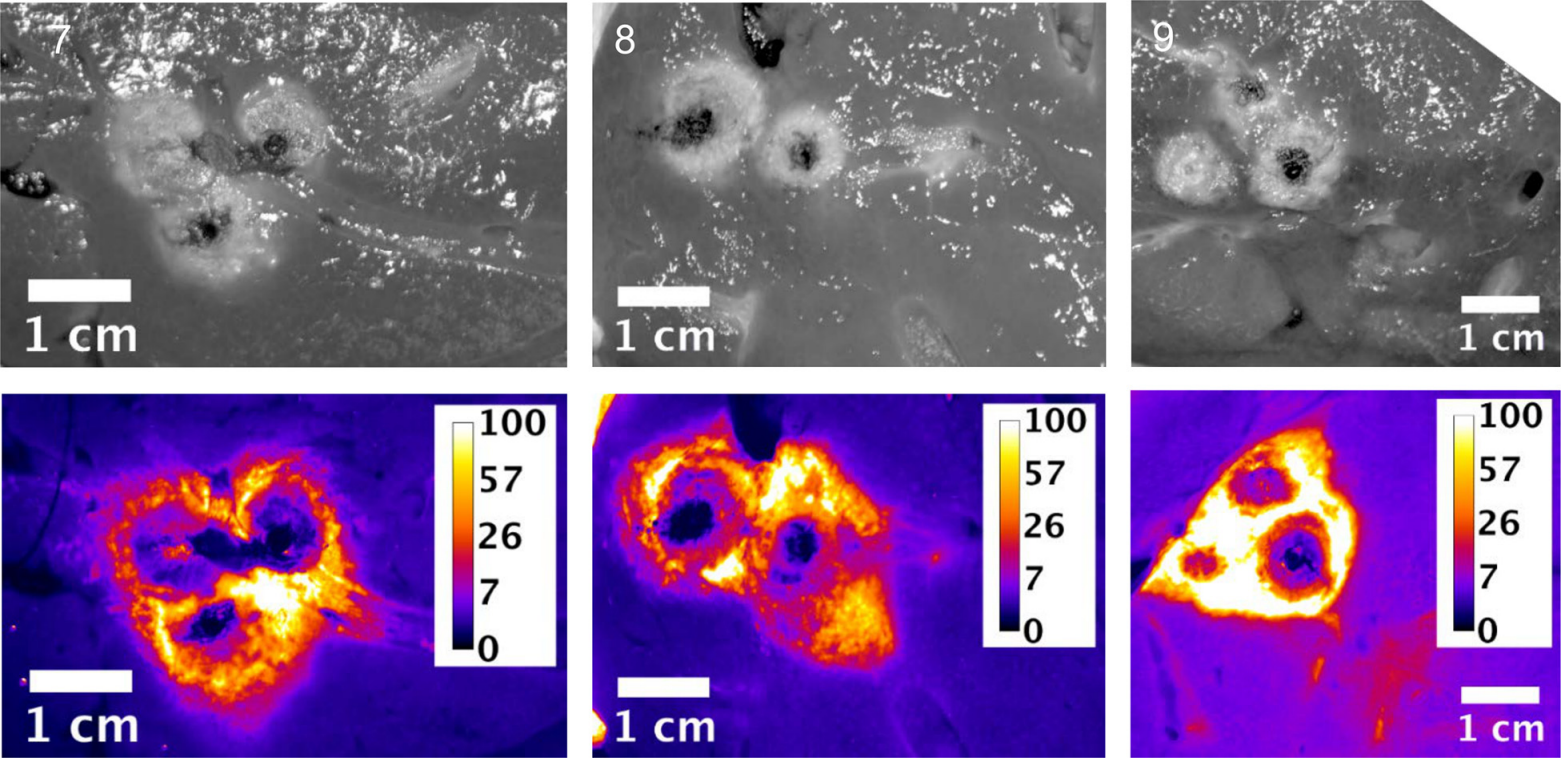
Plain and fluorescent images of ablation zone in pigs in Study B, group 2. Each pig received three sequential, overlapping 12 minute ablations and 3 minute cool down between ablations (total time = 45 minutes) with the Covidien device. Scale is μg/g doxorubicin.

**Fig 5:**
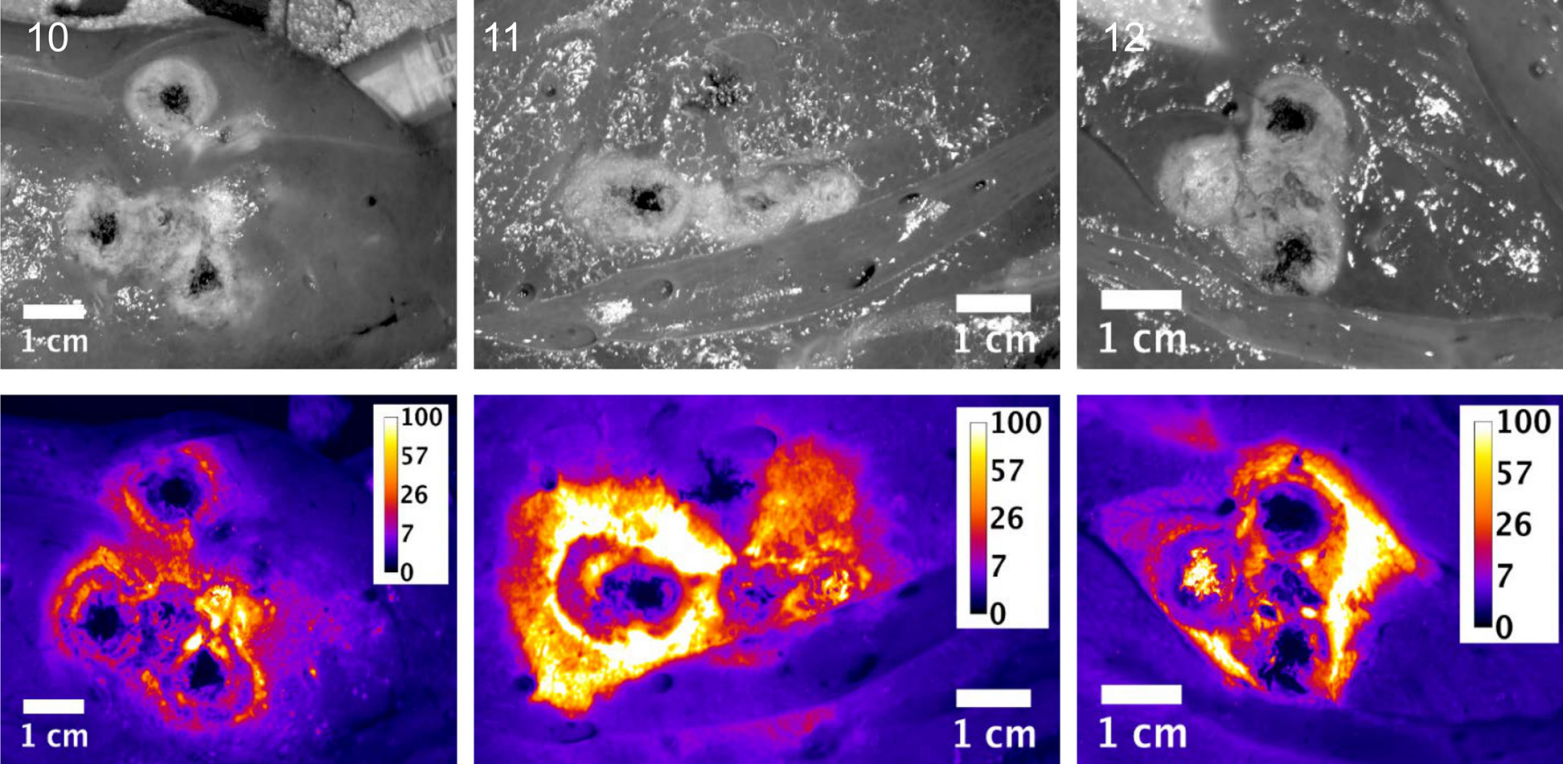
Plain and fluorescent images of ablation zone in pigs in Study B, group 3. Each pig received six sequential, overlapping 12 minute ablations and 3 minute cool down between ablations (total time = 90 minutes) with the Covidien device. Scale is μg/g doxorubicin.

Slices of the ablation zones were also imaged for fluorescent signal attributed to free doxorubicin. Figs 3, 4 and 5 show plain and fluorescent images of the ablations zones from pigs in groups 1, 2 and 3 respectively. The actual ablation zone surrounding the probe tracks shows coagulation necrosis. This tissue is non-viable and is not perfused, thus doxorubicin from LTLD does not diffuse into these areas and these areas are dark in the fluorescent images. When used with RFA, intravenously administered LTLD takes advantage of the tissue surrounding the ablation where the temperature exceeds 40°C but is still well perfused and viable, resulting in triggered intravascular release (TIR) of drug in the tumor margins where micrometastases may occur.

As indicated in the tissue samples analyzed by LC/MS, the fluorescent intensity is highest within a first ~1 cm margin around the ablation zone. In all cases, doxorubicin concentration approaching 100 μg/g concentration in various regions around the ablation zone was observed, although with some asymmetry to the doxorubicin deposition.

Since the LC/MS measurements represent the average concentration of punch biopsy tissue samples 8 mm in diameter and 1 cm long and due to the large concentration gradients, it is reasonable that the LC/MS concentrations are considerably smaller than the maximal concentrations observed in fluorescent image data.

**Fig 6:**
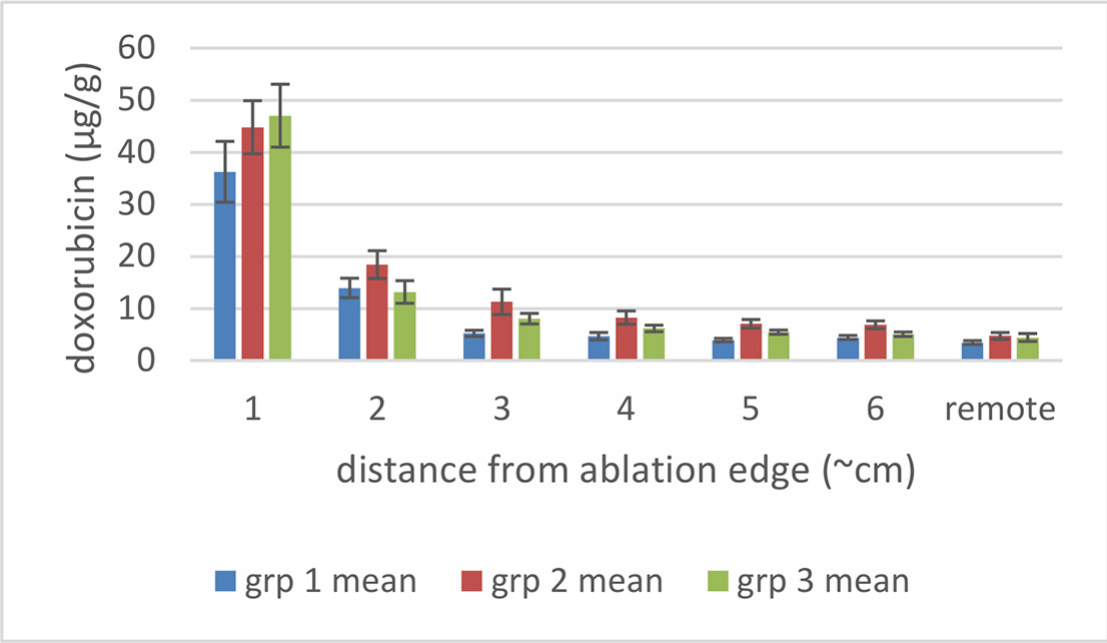
Mean (± SEM) doxorubicin tissue concentrations around the ablation zones of pigs in Study C. Punch biopsies were collected radiating out from the liver ablation zone after 1 (group 1, n = 3), 3 (group 2, n = 3) or 6 (group 3, n = 3) sequential, overlapping ablations using the Angiodynamics device. Distance 1 is just adjacent to the ablation margin and distance 6 is the furthest away from the ablation margin.

In Study C, animals were treated as in Study B, but with an Angiodynamics generator and electrodes. The results and conclusions were similar to those in study B, although the concentrations closest to the ablation margin were higher (~40–50 μg/g) than in study B, perhaps due to probe geometry (Fig 6).

**Fig 7:**
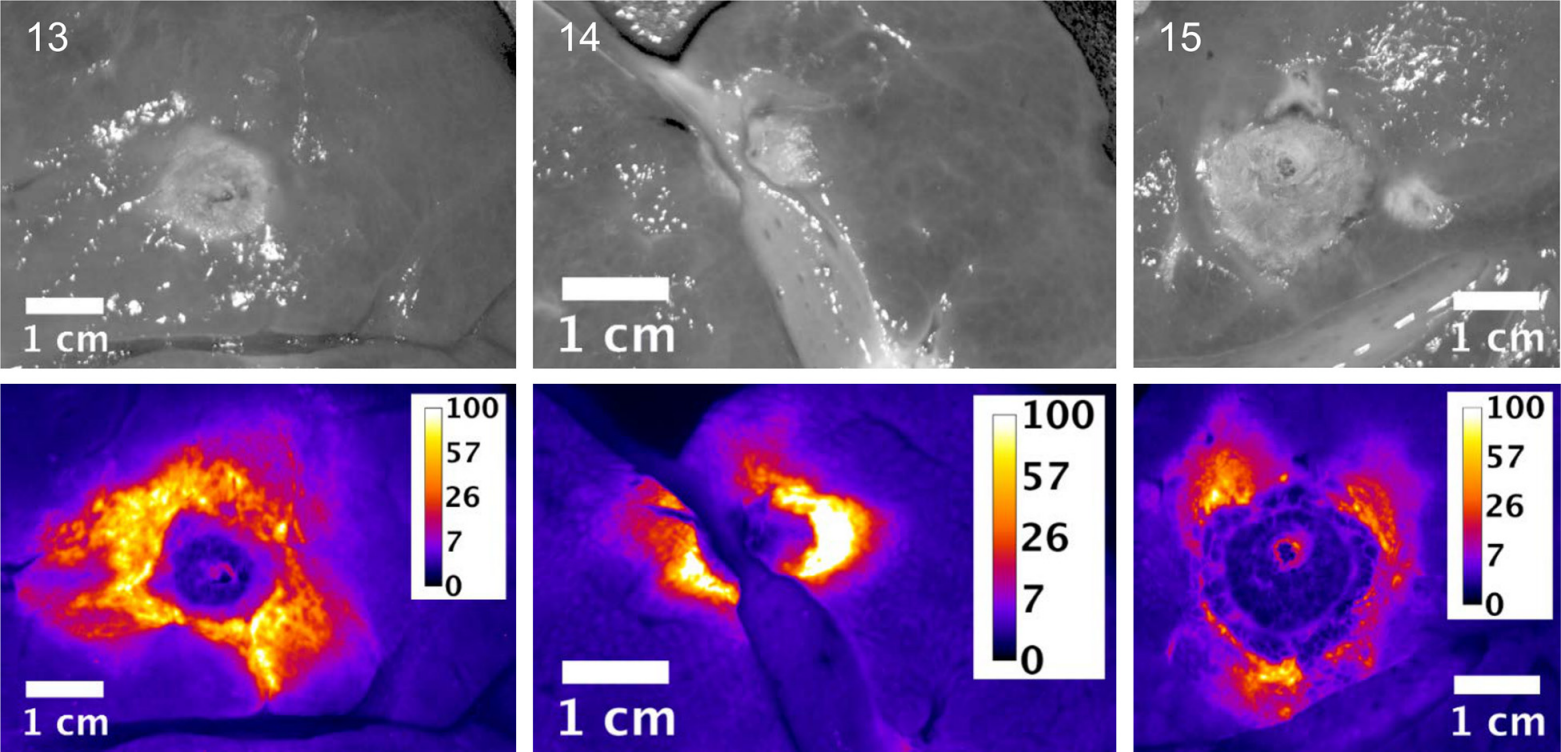
Plain and fluorescent images of ablation zone in pigs in Study C, group 1. Each pig received a single 12 minutes ablation and 3 minute cool down (total time = 15 minutes) with the Angiodynamics device. Scale is μg/g doxorubicin.

**Fig 8:**
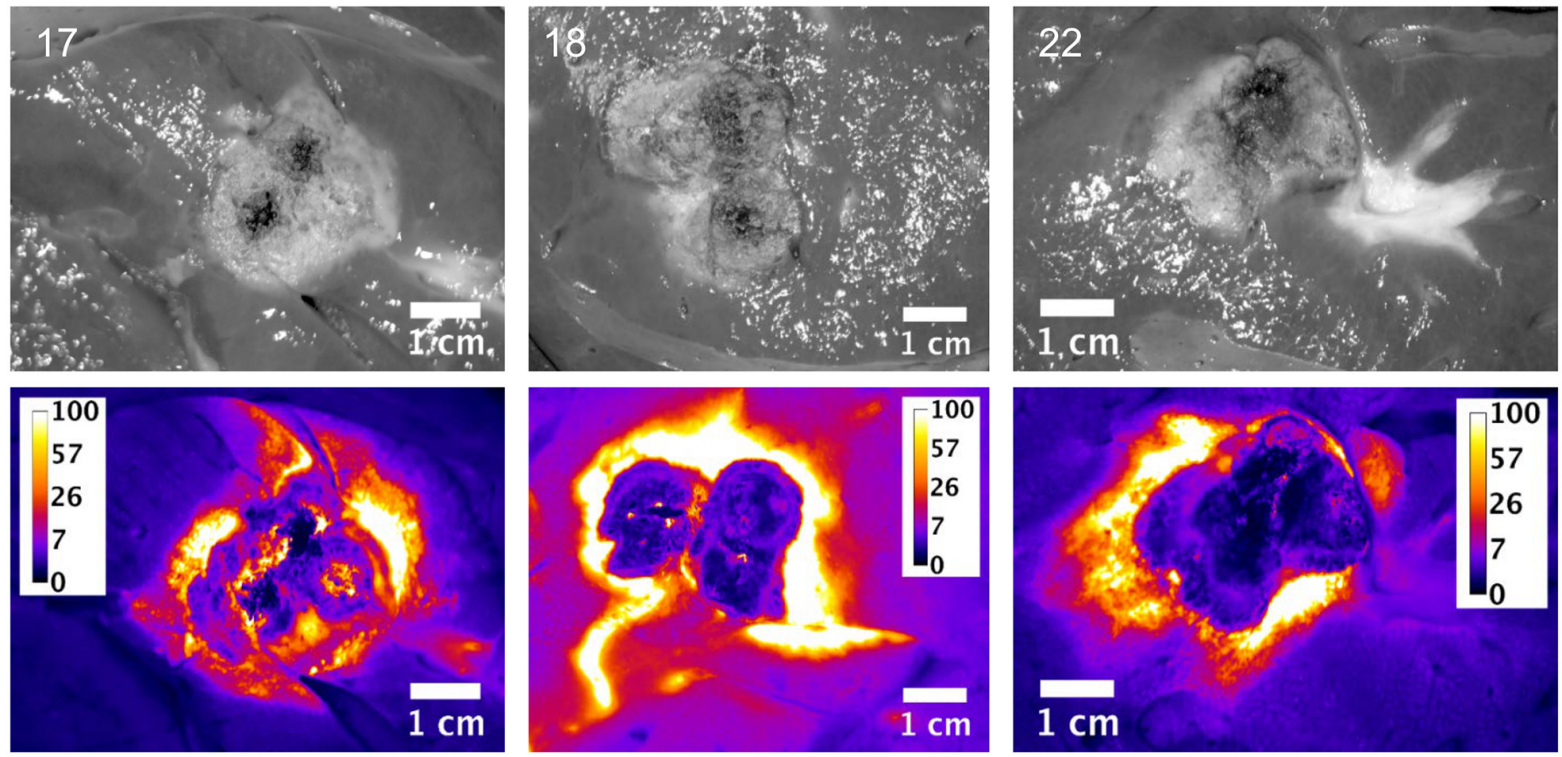
Plain and fluorescent images of ablation zone in pigs in Study C group 2. Each pig received three sequential, overlapping 12 minute ablations and 3 minute cool down between ablations (total time = 45 minutes) with the Angiodynamics device. Scale is μg/g doxorubicin.

**Fig 9:**
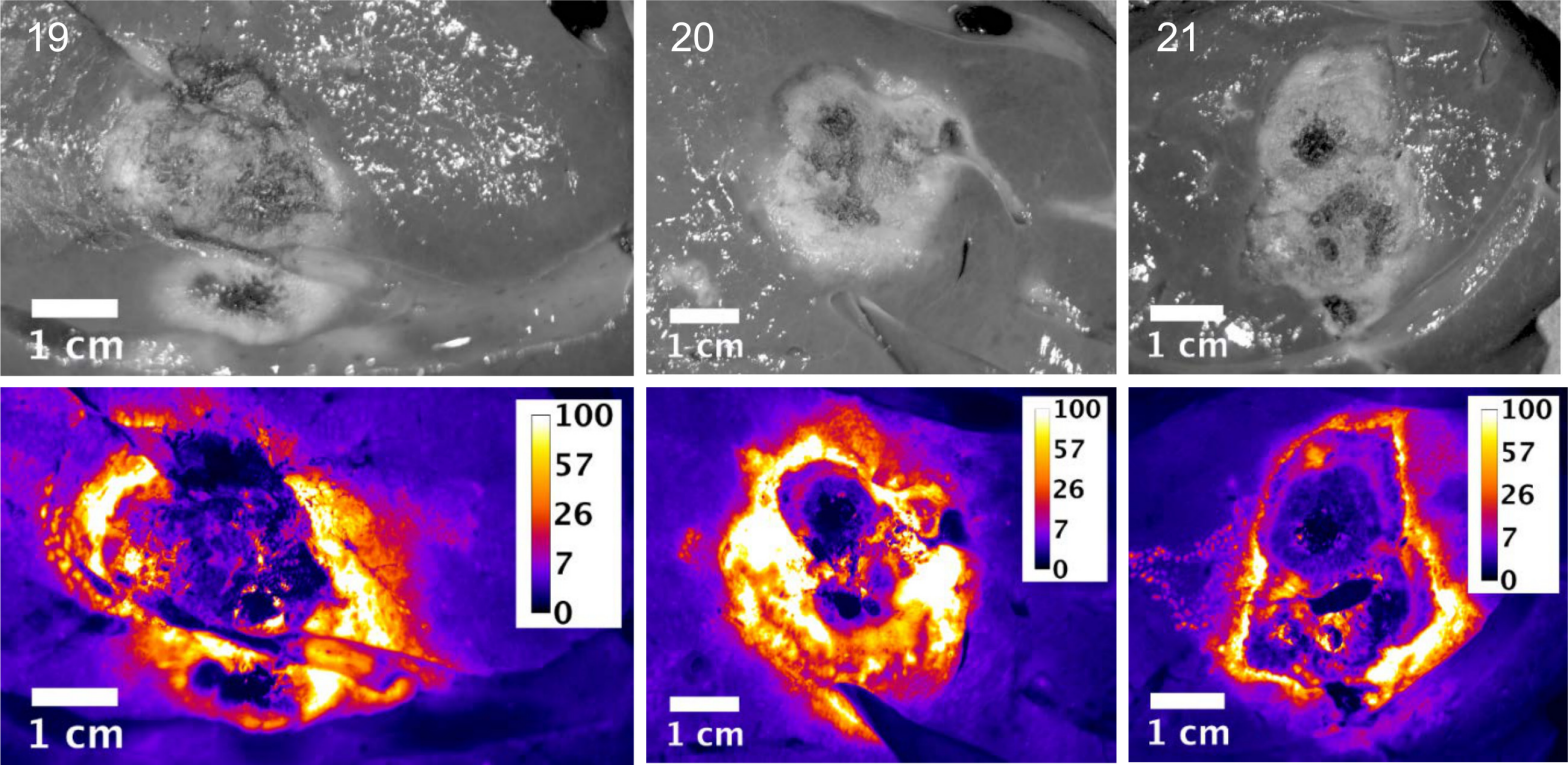
Plain and fluorescent images of ablation zone in pigs in Study C, group 3. Each pig received six sequential, overlapping 12 minute ablations and 3 minute cool down between ablations (total time = 90 minutes) with the Angiodynamics device. Scale is μg/g doxorubicin.

Slices of the ablation zones were again imaged for fluorescent signal attributed to free doxorubicin. Figs 7, 8 and 9 show white light photographic and fluorescent images for the ablation zones from pigs in groups 1, 2 and 3 respectively. As indicated in the tissue samples analyzed by LC/MS, the fluorescent intensity is maximal within the first 1 cm margin around the ablation zone. In all cases, enhanced doxorubicin concentration around the ablation zone is observed, although with some asymmetry to the doxorubicin deposition.

**Fig 10:**
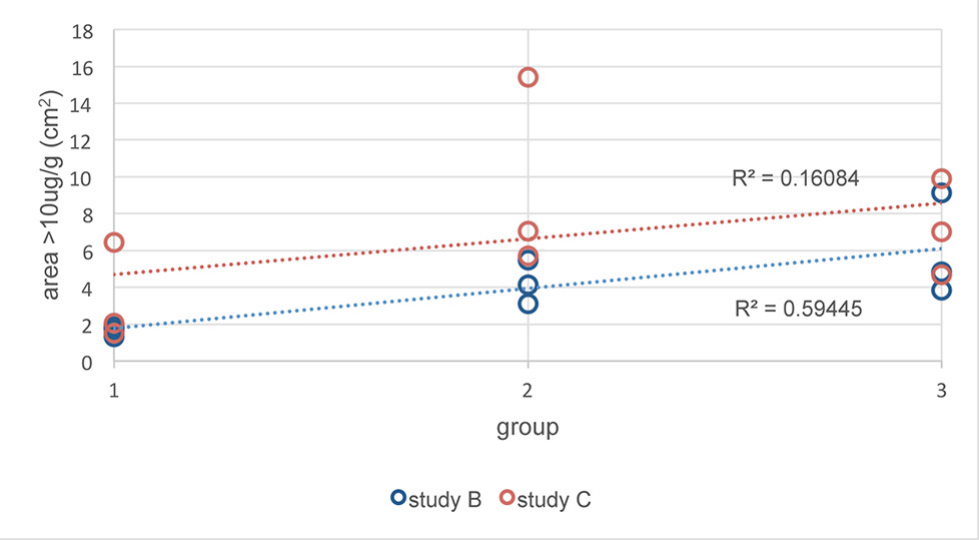
Total areas of the ablation zones with concentrations of doxorubicin above 10 μg/g. Concentration was determined by fluorescent imaging in groups 1 (single ablation), 2 (3 overlapping ablations) and 3 (6 overlapping ablations) in study B (Covidien device) and C (Angiodynamics device).

A histogram of the pixel intensities in the region of interest (the ablation zone) of each image in studies B and C was generated and the intensity values were converted to concentrations based on the calibration curve. The total area with doxorubicin concentrations above 10 μg/g was calculated for each ablation zone in each pig and is shown graphically in Fig 10. There is a trend for increasing area above 10 μg/g in both studies between group 1 (single ablation) and groups 2 and 3 (3 and 6 overlapping ablations), but this only reaches statistical significance in study B (Covidien device).

The mean probe temperature at one minute after the end of each ablation with the Covidien “cool tip” probe was 40.5°C (±3.1°C) while the high mean probe temperature at one minute after the end of each ablation with the Angiodynamic probe was 62.3°C (±6.5°C).

## Discussion

In nonclinical studies, LTLD plus mild hyperthermia (thermal dose 42°C for 1 hour to the tumor site) demonstrated higher tumor drug concentrations and superior anti-tumor effects against human squamous cell, ovarian, prostate, and colon xenografts and against a mouse mammary carcinoma when compared to non-thermo sensitive doxorubicin-containing liposomes, LTLD without heat, heat alone or saline treated controls [22–24]. LTLD has also shown increased doxorubicin concentrations in the targeted tissue after mild hyperthermia using high intensity focused ultrasound (reviewed in [25]). Here, LTLD was combined with RFA with the goal of delivering high concentrations of doxorubicin within the margin of the ablation zone, where the temperatures (40–50°C) are insufficient to kill tumor cells, but adequate to release drug from LTLD as shown in computer-simulation studies [6].

Our study did not evaluate the pharmacokinetics of doxorubicin after LTLD administration extensively, but other theoretical [26] and empirical [27] studies have shown that localized hyperthermia does not affect the overall blood circulation or organ accumulation of various thermally sensitive liposomal doxorubicin formulations. Similarly, studies of LTLD and free doxorubicin in rabbits with and without hyperthermia have shown that the tissue concentrations of doxorubicin were very similar (with the exception of the liver, which is known to take up liposomes) after LTLD (without HIFU) or free doxorubicin. The tissue concentrations of doxorubicin were very similar (with the exception of the heated tumor and the lung) after LTLD with or without hyperthermia [28, 29]. In the current study, doxorubicin concentrations of ~5 μg/g were found in “remote” liver samples that did not receive any hyperthermia.

The RF power delivered via the electrode decreases in proportion to the square of the distance from the electrode [30]. Therefore, regions not in the immediate area of the electrode are not affected. Doxorubicin concentrations in remote areas of the liver were ~5μg/g which is in the range of 50% growth inhibitory concentrations for 13 different hepatocellular carcinoma cell lines (0.14–3.5 μg/g [31]) and so these tissues may show a transient decrease in growth after LTLD administration.

Typically liver tumors less than 3 cm in diameter can be completely ablated, including a 1 cm tumor-free safety margin around the tumor (as determined by imaging). Thus a total ablation diameter of 5 cm is required for a 3 cm tumor. This practice of obtaining a 1 cm margin of tumor-free tissue parallels the surgical method of liver tumor resection [30, 31] and studies have demonstrated increased local recurrence rates for margins less than 1 cm [32]. For tumors greater than 3 cm in diameter, there is a greater propensity to leave viable tumor cells in the margins or clefts of viable tissue between overlapping ablation zones. This increases the likelihood of recurrence at the site of the original tumor as well as elsewhere within the liver at sites remote from the treatment area (due to vascular spread) [33].

The concept behind the treatment approach of utilizing the concurrent administration of LTLD with RFA is to improve the overall therapeutic benefit of RFA by creating a large concentration gradient of doxorubicin in the immediate region of the tumor bordering the zone of RFA-induced cell necrosis. The temperature isotherms produced in this boundary region should be adequate to activate doxorubicin release by the thermally sensitive liposomes in the intravascular space around the ablation zone. This process increases the region of tissue that can be treated beyond that achievable by use of RFA alone and deposits doxorubicin in clefts of viable tissue between ablations. The current study did not record temperatures in and around the probe during the ablation procedure, although temperatures of 40° - 62°C were recorded at the end of each ablation which is in agreement with computer simulation studies of RFA in tumor tissue that have demonstrated hyperthermic zones around the ablation zones with temperatures between 40°C and 50°C [6].

These simulation studies of the combination of RFA and LTLD or conventional doxorubicin in the treatment of liver tumors have shown that LTLD treatment resulted in tumor tissue drug concentrations of ~9.3 μg/g with the highest values within 1 cm outside the ablation zone boundary. Free doxorubicin treatment resulted in relatively uniform tissue concentrations of ~3.0 μg/g throughout [6]. Our empirical studies support the theoretical model in that high concentrations of doxorubicin were deposited in the tissue just outside the ablation zones. “Remote” samples of the liver (in areas far from the ablation and therefore not subject to hyperthermia) in our study were consistently about 5 μg/g. The absolute concentration values measured in the current study were considerably larger than in this prior modelling study, possibly in part due to the fact that normal liver tissue (vessel structure, stromal cells, interstitial pressure etc.,) is very different from tumor tissue and takes up doxorubicin very rapidly [34] compared to the tumor cells considered in the computer model [6, 35]. The doxorubicin concentrations were consistently increased in the surrounding tissue volume, however with asymmetric distribution, most likely associated with variations in vascularity of liver tissue. The fluorescent images provide a higher resolution picture of the actual doxorubicin concentration gradients surrounding the ablation zone (Figs 3–5 and Figs 7–9). In contrast, the punch biopsies samples selected radial trajectories, and coupled with the reduced geometric resolution resultant of the 8 mm diameters used, represent average tissue concentrations around the ablation zone. The current study demonstrates that increasing ablation duration by a factor of 3–6 (13 min to 39 or 73 minutes) increases the local concentration as well as the tissue volume exposed to drug. Further, doxorubicin delivery is extended further from the ablation zone margin, beyond 1 cm, when multiple ablations were used (Figs 2 and 6). Both effects are considered to increase the potential therapeutic benefit of the clinical combination of RFA and LTLD. Finally, the effects were demonstrated with two commercially available, clinical RFA devices in healthy pig livers and results are supported by both tissue extraction assay and fluorescent imaging. Our finding that longer duration ablations result in greater and broader deposition of doxorubicin may help to understand the findings in a recent phase III study of ThermoDox plus RFA in primary liver cancer in which a post-hoc analysis found that patients treated with ThermoDox and RFA lasting 45 minutes or longer had a greater overall survival than those treated for less than 45 minutes [17].

## Supporting Information

S1 Table Premedication and anesthesia medications. (DOCX)

S1 Fig Dissection of ablation zone (3 overlapping ablations). (TIF)
