# Supplementary material for: Increased Duration of Heating Boosts Local Drug Deposition during Radiofrequency Ablation in Combination with Thermally Sensitive Liposomes (ThermoDox) in a Porcine Model
Source: PLoS One. 2015 Oct 2;10(10):e0139752. doi: 10.1371/journal.pone.0139752 (PMC4592068; doi:10.1371/journal.pone.0139752)
Supplement: S1 Table — (DOCX) [file pone.0139752.s001.docx]

Table S1: Premedication and anesthesia medications

| Medication | Dose | Route | Time |
| --- | --- | --- | --- |
| DepoMedrol | 1-5 mg/kg | IM | ~24 hr prior to surgery |
| Methylpredisolone | 3 mg/kg | IV | Prior to drug infusion |
| Diphenhydramine | 1 mg/kg | IV | Prior to drug infusion |
| Ranitidine | 1 mg/kg | IV | Prior to drug infusion |
| Telazol | 2.0-8.8 mg/kg | IM | Pre-induction |
| Atropine | 0.03-0.05 mg/kg | IM | Pre-induction |
| Fentanyl | 5-30 µg/kg | IV | Induction |
| Benzodiazepine | 0.1-0.5 mg/kg | IV | Induction |
| Isoflurane | 0.5-5% | inhalation | Anesthesia maintenance |
| Fentanyl | 10-100 µg/kg | Constant infusion | Anesthesia maintenance |
| Propofol | 1-2 mg/kg | Constant infusion | Anesthesia maintenance |
