# Supplementary figures and images for: Increased Duration of Heating Boosts Local Drug Deposition during Radiofrequency Ablation in Combination with Thermally Sensitive Liposomes (ThermoDox) in a Porcine Model
Source: PLoS One. 2015 Oct 2;10(10):e0139752. doi: 10.1371/journal.pone.0139752 (PMC4592068; doi:10.1371/journal.pone.0139752)

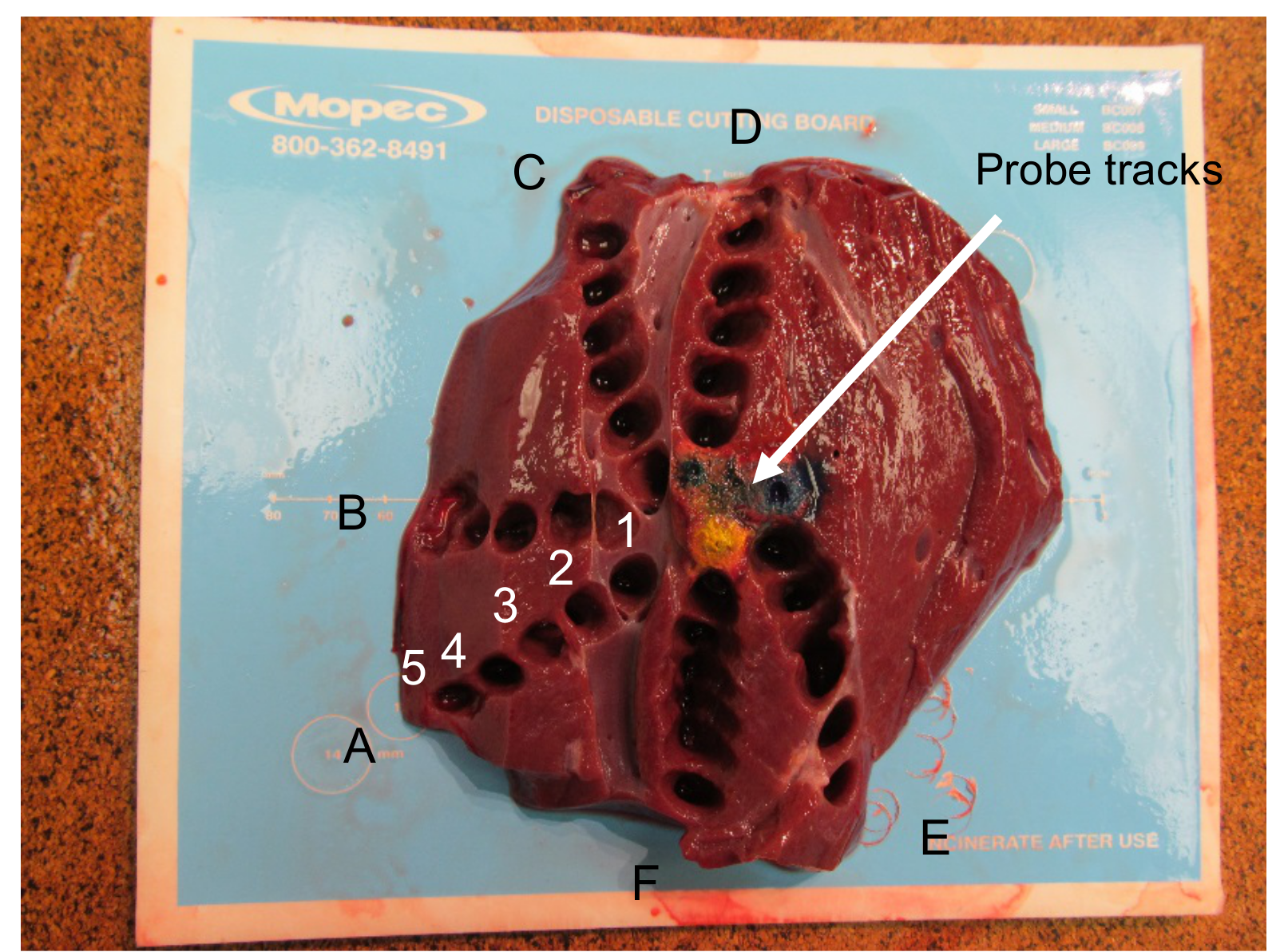

Supplement: S1 Fig — (TIF) [file pone.0139752.s002.tif]
